# *Streptococcus mutans* adhesion force sensing in multi-species oral biofilms

**DOI:** 10.1038/s41522-020-0135-0

**Published:** 2020-06-24

**Authors:** Can Wang, Henny C. van der Mei, Henk J. Busscher, Yijin Ren

**Affiliations:** 1 Department of Orthodontics, W.J. Kolff Institute, University of Groningen and University Medical Center Groningen, Groningen, The Netherlands; 2 Department of Biomedical Engineering, W.J. Kolff Institute, University of Groningen and University Medical Center Groningen, Groningen, The Netherlands

**Keywords:** Biofilms, Dentistry

## Abstract

Bacteria utilize chemical and mechanical mechanisms to sense their environment, to survive hostile conditions. In mechanical sensing, intra-bilayer pressure profiles change due to deformation induced by the adhesion forces bacteria experience on a surface. Emergent properties in mono-species *Streptococcus mutans* biofilms, such as extracellular matrix production, depend on the adhesion forces that streptococci sense. Here we determined whether and how salivary-conditioning film (SCF) adsorption and the multi-species nature of oral biofilm influence adhesion force sensing and associated gene expression by *S. mutans*. Hereto, *Streptococcus oralis*, *Actinomyces naeslundii*, and *S. mutans* were grown together on different surfaces in the absence and presence of an adsorbed SCF. Atomic force microscopy and RT-qPCR were used to measure *S. mutans* adhesion forces and gene expressions. Upon SCF adsorption, stationary adhesion forces decreased on a hydrophobic and increased on a hydrophilic surface to around 8 nN. Optical coherence tomography showed that triple-species biofilms on SCF-coated surfaces with dead *S. oralis* adhered weakly and often detached as a contiguous sheet. Concurrently, *S. mutans* displayed no differential adhesion force sensing on SCF-coated surfaces in the triple-species biofilms with dead *S. oralis*, but once live *S. oralis* were present *S. mutans* adhesion force sensing and gene expression ranked similar as on surfaces in the absence of an adsorbed SCF. Concluding, live *S. oralis* may enzymatically degrade SCF components to facilitate direct contact of biofilm inhabitants with surfaces and allow *S. mutans* adhesion force sensing of underlying surfaces to define its appropriate adaptive response. This represents a new function of initial colonizers in multi-species oral biofilms.

## Introduction

Environmental sensing is of vital importance for bacteria to counter the many hostile conditions they may endure during their growth. Bacteria can sense and respond to chemical signals such as pH, ionic strength, or auto-inducers involved in quorum sensing^1^. However, bacteria also respond to a variety of mechanical signals^2^, such as adhesion forces arising from a surface to which they adhere. Upon adhesion to a surface, bacterial cell surfaces slightly deform under the influence of the adhesion forces^3^, which impacts the intra-bilayer pressure profile across their lipid membrane^4^. These pressure profile changes can stimulate gene expressions in bacteria upon adhesion to a surface that are important for the adaptation of planktonically growing bacteria into a biofilm mode of growth. In a biofilm mode of growth, biofilm inhabitants often possess new, emergent properties alien to their planktonic counterparts^5^. Increasing adhesion forces of *Staphylococcus aureus* on surfaces decreased expression of *icaA* genes and production of poly-*N*-acetylglucosamine and eDNA, both of direct relevance as a glue in the formation of the extracellular polymeric substance (EPS) matrix of a biofilm^6^. Also, NsaS sensor and NsaAB efflux pump transcript levels in *S. aureus* were enhanced as a response to antibiotic presence when staphylococci were adhering to a surface^7^ *Streptococcus mutans*, one of the main cariogenic strains in the oral cavity, has been demonstrated to possess three adhesion force sensitive genes (*brpA*, *gbpB*, *comDE*) responsible for emergent EPS production on silicone rubber, bacterial grade polystyrene, tissue-grade polystyrene, and glass^8^ (these surfaces are listed in the order of decreasing water contact angle (“hydrophobicity”), corresponding with a decrease in adhesion forces from 20 nN to 3 nN). Adhesion force-induced gene expression and emergent EPS production in *S. mutans* biofilms were limited to the first 20–30 µm above a substratum surface, but fully absent in a quorum-sensing-deficient *S. mutans* strain. A distance of 20–30 µm above a substratum surface is within the “calling distances” reported for producing, releasing, sensing, and responding to auto-transducer gradients in quorum sensing^9–11^. Collectively, this suggests that the results of adhesion force sensing by streptococci directly in contact with a surface are carried into a biofilm through quorum sensing. This highlights the interplay between chemical and mechanical environmental sensing by adhering bacteria and their role in biofilm formation.

In oral biofilm formation, adsorption of salivary proteins precedes adhesion of initial bacterial colonizers to a surface, modulating bacterial adhesion to surfaces^12^ and possibly adhesion force sensing and associated surface adaptation. Moreover, oral biofilms are composed of many different bacterial strains and species that are all involved in biofilm formation in a spatio-temporal sequence^13,14^. *Streptococcus oralis* and *Actinomyces naeslundii* have their own role in this sequence and are among the dominant initial colonizers that adhere to the adsorbed salivary-conditioning film (SCF) and form a co-aggregating pair. After adhesion of initial colonizers, later colonizers, such as *S. mutans* and other oral bacterial strains, come into play due to co-aggregation^13^ or local changes in the environment^15,16^, giving them a different position in the spatio-temporal sequence of oral biofilm formation. Oral bacteria not only adhere to tooth surfaces but also to the surfaces of composite restorations, orthodontic appliances^17^, and other oral bacterial cell surfaces. Accordingly, oral bacteria encounter many different surfaces, each with their own surface charge and hydrophobicity, to which they may adhere and adapt with different emergent properties^11^.

The aim of this article is to determine whether and how SCF adsorption and the presence of *S. oralis* and *A. naeslundii* influence adhesion force sensing and associated expression of *brpA*, *gbpB*, and *comDE* by *S. mutans* in a triple-species, oral biofilm model. To evaluate possible interference by *S. oralis* or *A. naeslundii* on *S. mutans* adaptive gene expression, live and dead *S. oralis* and/or *A. naeslundii* were used to grow triple-species biofilms. Single bacterial contact probe atomic force microscopy (AFM) was applied to measure the adhesion forces that *S. mutans* senses on different surfaces in the absence and presence of an adsorbed conditioning film, while adaptive gene expressions in biofilms were evaluated using reverse transcription-quantitative PCR (RT-qPCR). Genes were selected that all have a demonstrated role in adhesion and growth of *S. mutans*: *brpA* regulates cell wall stress responses, biofilm cohesiveness, and biofilm formation, whereas *gbpB* regulates antibiotic sensitivity, osmotic and oxidative stresses, cell wall construction and maintenance, cell shape, hydrophobicity, and sucrose-dependent biofilm formation, and *comDE* relates with persister cell formation and bacteriocin production^8^.

## Results

### *S. mutans* adhesion forces

*S. mutans* adhesion forces on different surfaces were measured using single bacterial contact probe AFM. The bacterial probe was brought to a surface and after different time periods retracted from the surface. During this bond maturation time period, bacterial cell surface appendages re-arrange, molecules change conformation, and water is removed from between the bacterial cell surface and a substratum surface to increase the adhesion force. These processes are all physico-chemical in nature and equally occur in bacteria as well as in inert polystyrene particles^18^. All *S. mutans* adhesion forces increased with increasing bond maturation times (Supplementary Fig. 1) between the bacterium and any surface, whether glass, silicone rubber or a bacterial cell surface. Accordingly, adhesion forces were plotted as a function of bond maturation time (Supplementary Fig. 2) and were fitted to an exponential function, yielding an initial adhesion force *F*_0_ measured immediately upon contact, a characteristic bond maturation time *τ*, and a stationary adhesion force *F*_stationary_ reached after complete bond maturation (see Supplementary Material for calculational details). All initial adhesion forces were significantly lower than the stationary adhesion forces calculated (Table 1). Initial adhesion forces increased fastest on hydrophilic glass surfaces (*τ* is 4 s), and slowest on hydrophobic silicone rubber surfaces (τ equals 21 s). *S. mutans* adhesion forces with any of the other two strains increased with intermediate bond maturation times. Stationary adhesion forces on glass increased after adsorption of a SCF (*P* > 0.05, Mann–Whitney test), whereas on silicone rubber stationary adhesion forces decreased (*P* < 0.05, Mann–Whitney test). Interestingly, both surfaces displayed similar stationary *S. mutans* adhesion forces after adsorption of a SCF (*P* > 0.05, Mann–Whitney test). Stationary adhesion forces between *S. mutans* and *S. oralis*, and *A. naesliundii* were all smaller than the adhesion forces arising from the substratum surfaces (*P* > 0.05, one-way analysis of variance (ANOVA)). Live and dead, heat-killed *S. oralis* and *A. naeslundii* presented the same stationary adhesion forces to *S. mutans*, indicating that heat killing did not affect the adhesive surface properties of the strains. This was confirmed by zeta potential measurements, showing similarly negative zeta potentials for live and dead bacteria (Supplementary Table 1, *P* > 0.05, Mann–Whitney test). Summarizing, *S. mutans* stationary adhesion forces differed greatly on silicone rubber and glass surfaces, but converged upon adsorption of a SCF to these surfaces. *S. mutans* stationary adhesion forces to *S. oralis* and *A. naeslundii* cells were smaller than to the materials surfaces, regardless whether these bacteria were dead or alive.

**Table 1:** Initial and stationary streptococcal adhesion forces *F*_0_ and *F*_stationary_, together with the characteristic bond maturation time constant τ for *S. mutans* UA159 with different substratum surfaces in the absence and presence of an adsorbed salivary-conditioning film (SCF) and with *S. oralis* J22 and *A. naeslundii* T14V-J1.

*S. mutans* UA159 adhesion to	*F*_0_ (nN)	*τ* (s)	*F*_stationary_ (nN)
Glass^a^	0.7 ± 0.1^b,c^	4 ± 3^c^	4.1 ± 1.3^c^
SCF-coated glass	0.4 ± 0.1	8 ± 4	7.9 ± 4.3
Silicone rubber^a^	1.2 ± 0.4^b^	21 ± 11	19 ± 14^b^
SCF-coated silicone rubber	0.3 ± 0.1^c^	14 ± 9	7.8 ± 2.6
Live *S. oralis*	0.4 ± 0.1	13 ± 3	2.8 ± 0.1
Dead *S. oralis*	0.5 ± 0.2	13 ± 5	3.4 ± 0.9
Live *A. naeslundii*	0.6 ± 0.3	11 ± 9	4.1 ± 1.5
Dead *A. naeslundii*	0.8 ± 0.3	9 ± 4	5.5 ± 1.1

± represent SDs over 35 force–distance curves.

The force–distance curves were recorded using three separately prepared bacterial probes, taken out of three different bacterial cultures, each measuring adhesion forces on seven different contact points and measuring five force–distance curves for on each contact point.

^a^Data taken from Wang et al.^8^.

^b^Significantly different from SCF-coated substrata (*P* < 0.05, Mann–Whitney test).

^c^Significantly different from corresponding data on silicone rubber (*P* < 0.05, Mann–Whitney test).

### (Co-)aggregation of different strain combinations

The adhesion forces measured using AFM occurred after forced contact between the interacting surfaces. In order to determine up to what extent the three strains can be expected to adhere to each other and sense adhesion forces exerted upon each other during adhesion and biofilm formation from suspension, a co-aggregation assay was carried out. Co-aggregation of the three bacterial strains was investigated using the well-established and generally-accepted, semi-quantitative assay proposed by Kolenbrander and Andersen^19^. In the assay, equal volumes of one or more of the bacterial suspensions are mixed. Aggregation of any of the bacterial strains in mono-species suspension in the absence of other strains did not occur (Table 2), regardless of whether bacteria were alive or dead. Also, *S. mutans* did not co-aggregate with *S. oralis* nor *A. naeslundii* in dual-species suspensions, also regardless of whether alive or dead. This implies that in triple-species biofilms, *S. mutans* is unlikely to sense any adhesion forces arising from either *S. oralis* or *A. naeslundii*. Both alive and dead *S. oralis* and *A. naeslundii* co-aggregated in dual-species suspensions, while also triple-species suspensions demonstrated clear co-aggregation.

**Table 2:** (Co-)aggregation scores^1^ of different combinations of *S. mutans* UA159, live or dead *S. oralis* J22, and *A. naeslundii* T14V-J1 in mono-, dual-, and triple-species suspensions.

*S. mutans*	*S. oralis*	*A. naeslundii*	Score^a^
Mono-species suspensions			
Live	–	–	0
–	Live	–	0
–	Dead	–	0
–	–	Live	0
–	–	Dead	0
Dual-species suspensions			
Live	Live	–	0
Live	Dead	–	0
Live	–	Live	0
Live	–	Dead	0
–	Live	Live	3
–	Live	Dead	2
–	Dead	Live	2
–	Dead	Dead	2
Triple-species suspensions			
Live	Live	Live	3
Live	Live	Dead	2
Live	Dead	Live	2
Live	Dead	Dead	2

Experiments were done in triplicate with separately cultured bacteria, yielding similar scores.

^a^Score 0: no change in turbidity and no visible co-aggregates; Score 1: weak co-aggregation with dispersed aggregates in a turbid background; Score 2: clearly visible, small co-aggregates, not settling immediately; Score 3: large settling co-aggregates, leaving a slightly turbid suspension; Score 4: maximum co-aggregation large co-aggregates settled immediately leaving a fully clear supernatant^19^.

### Thickness and composition of biofilms

**Fig. 1:**
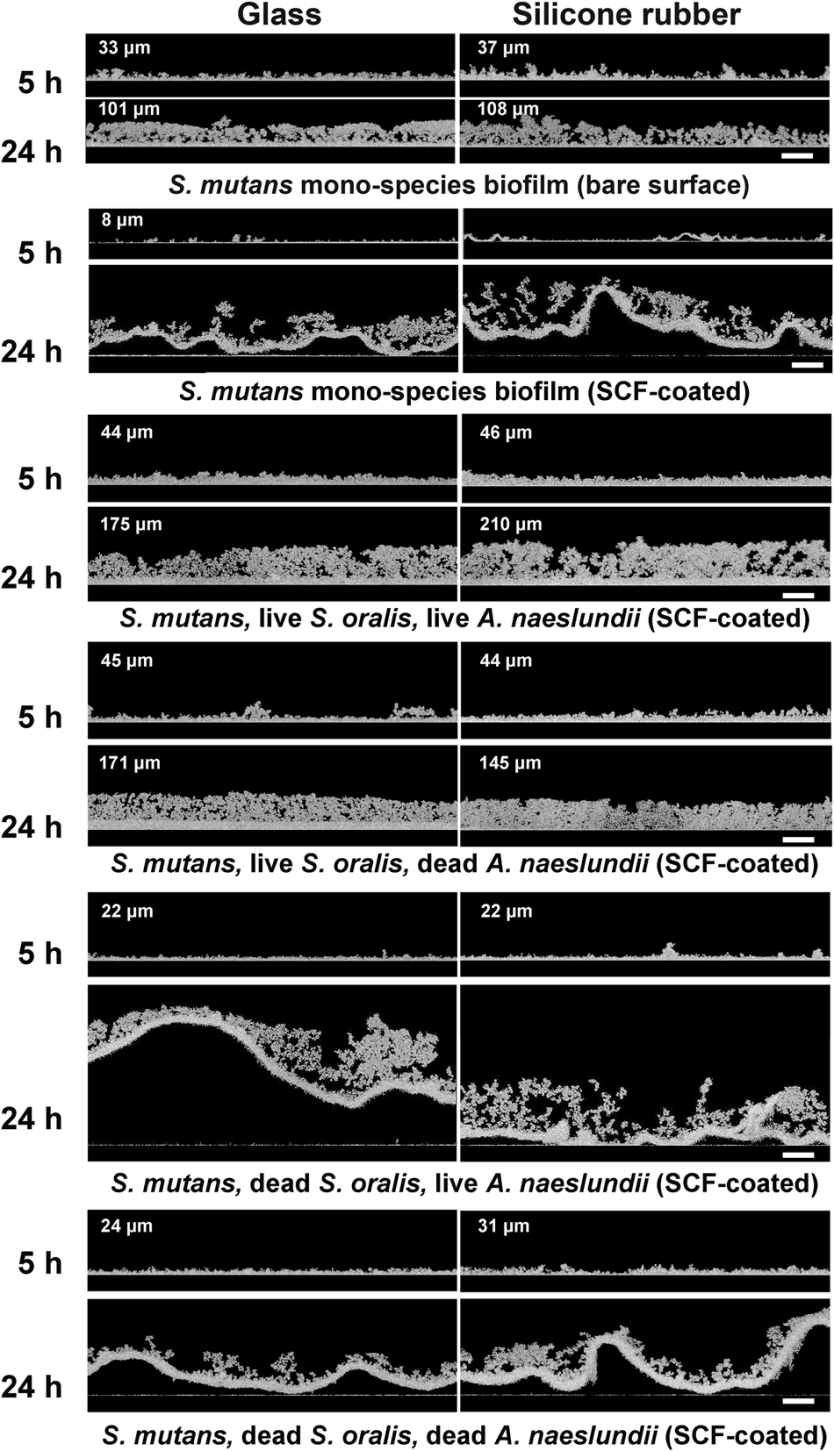
Examples of OCT images of *S. mutans* UA159 mono-species biofilm and different triple-species biofilms. The triple-species biofilms included live or dead *S. oralis* J22 and *A. naeslundii* T14V-J1 in addition to live *S. mutans* UA159 after 5 h and 24 h growth on different substratum surfaces. OCT images of *S. mutans* mono-species biofilms on glass and silicone rubber surfaces in the absence of an adsorbed salivary-conditioning film (SCF) are taken from Wang et al.^8^. Scale bars indicate 50 µm.

*S. mutans* mono-species biofilms and different triple-species biofilms were grown on SCF-coated glass and silicone rubber surfaces for 5 h and 24 h. To determine their thickness, biofilms were imaged using optical coherence tomography (OCT) (Fig. 1). Thickness could only be measured in cases of bacteria initially adhering to substratum surfaces in the absence of a SCF and in cases where live *S. oralis* were present. All 24 h biofilms were thicker than 5 h biofilms. Contiguous sheets of *S. mutans* mono-species biofilms and of triple-species biofilms in the absence of live *S. oralis* detached during handling of the biofilms from SCF-coated substrata, as caused by even the slightest fluid flow within the wells. This may well be due to the relatively small adhesion forces of the initial colonizers to SCFs as compared to bare substratum surfaces^20^. Moreover, it indicates that only live *S. oralis* paves a way through the SCF to ensure direct, strong contact of individual biofilm inhabitants and strong adhesion of an entire triple-species biofilm to a substratum surface.

**Fig. 2:**
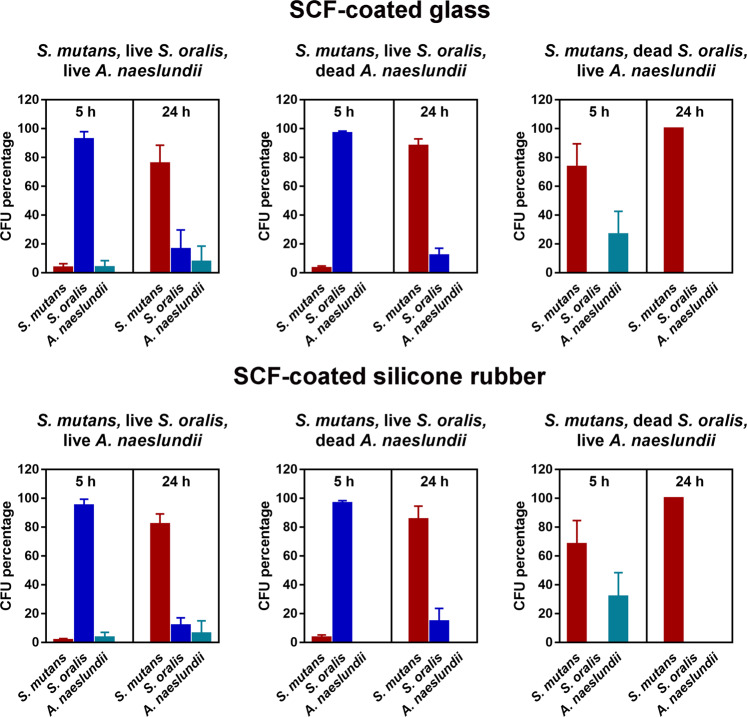
The percentage composition of different triple-species biofilms containing *S. mutans* UA159, in combination with live or dead *S. oralis* J22 and *A. naeslundii* T14V-J1. The percentage composition was expressed relative to the total number of CFU/cm^2^ in a biofilm. Error bars represent SDs over triplicate experiments with separate bacterial cultures.

To monitor the composition of the triple-species biofilms grown as a function of time, biofilms were scraped off the surfaces, suspended in buffer and serial dilutions grown on different (selective) agar plates. Growth on blood agar allowed growth of all strains, whereas selective agar plates only allowed growth of exclusively *S. mutans* or *A. naeslundii*. *S. oralis* counts were then determined by subtraction from the blood agar count. *S. mutans* mono-species biofilms contained between 4.3 × 10^7^ CFU/cm^2^ and 9.4 × 10^7^ CFU/cm^2^ after 5 h of growth on SCFs adsorbed on glass and silicone rubber surfaces, respectively. *S. mutans* numbers increased to 19.0 × 10^7^ CFU/cm^2^ and 16.3 × 10^7^ CFU/cm^2^ after 24 h of growth (Table 3). The percentage composition of each strain in different biofilms was calculated by dividing the number of CFU of each strain by the total number of CFUs in a biofilm, as displayed in Fig. 2. Biofilm compositions were similar on SCF-coated glass and SCF-coated silicone rubber. Whenever present as a live organism, *S. oralis* was the dominant strain in 5 h-old triple-species biofilms, whereas in corresponding 24 h-old biofilms *S. mutans* became the dominant strain. *A. naeslundii* presence remained low, except when grown in triple-species with dead *S. oralis*.

**Table 3:** The number of CFU per unit substratum surface area (10^7^/cm^2^) of each strain in *S. mutans* UA159 mono-species and in different triple-species biofilms with live or dead *S. oralis* J22 and *A. naeslundii* T14V-J1 on salivary conditioning film (SCF)-coated glass and silicone rubber surfaces.

Biofilm	Biofilm inhabitants	SCF-coated glass		SCF-coated silicone rubber	
		5 h	24 h	5 h	24 h
Mono-species	Live *S. mutans*	4.3 ± 0.6	19.0 ± 6.3	9.4 ± 2.4	16.3 ± 6.6
Triple-species	Live *S. mutans*	1.5 ± 0.3	4.6 ± 2.7	1.8 ± 0.3	6.3 ± 3.1
	Live *S. oralis*	33.4 ± 7.2	0.1 ± 0.1	41.6 ± 0.3	0.3 ± 0.1
	Live *A. naeslundii*	5.2 ± 1.9	0.1 ± 0.0	5.4 ± 3.3	0.1 ± 0.1
Triple-species	Live *S. mutans*	4.1 ± 1.2	17.3 ± 3.5	3.5 ± 0.8	15.4 ± 3.2
	Dead *S. oralis*	0	0	0	0
	Dead *A. naeslundii*	0	0	0	0
Triple-species	Live *S. mutans*	1.1 ± 0.5	0.5 ± 0.1	0.8 ± 0.3	0.3 ± 0.1
	Live *S. oralis*	35.5 ± 5.8	0.1 ± 0.0	28.9 ± 13.4	0.1 ± 0.04
	Dead *A. naeslundii*	0	0	0	0
Triple-species	Live *S. mutans*	6.6 ± 1	10.0 ± 0.4	3.6 ± 1.1	10.0 ± 5.6
	Dead *S. oralis*	0	0	0	0
	Live *A. naeslundii*	2.8 ± 2.1	ND^a^	2.0 ± 1.2	ND^a^

The number of CFU per unit substratum surface area.

Experiments were done in triplicate with separately cultured bacteria. ± represent SDs.

^a^ND is not detectable CFU < 10^3^/cm^2^.

### *S. mutans* gene expressions in different biofilms

**Fig. 3:**
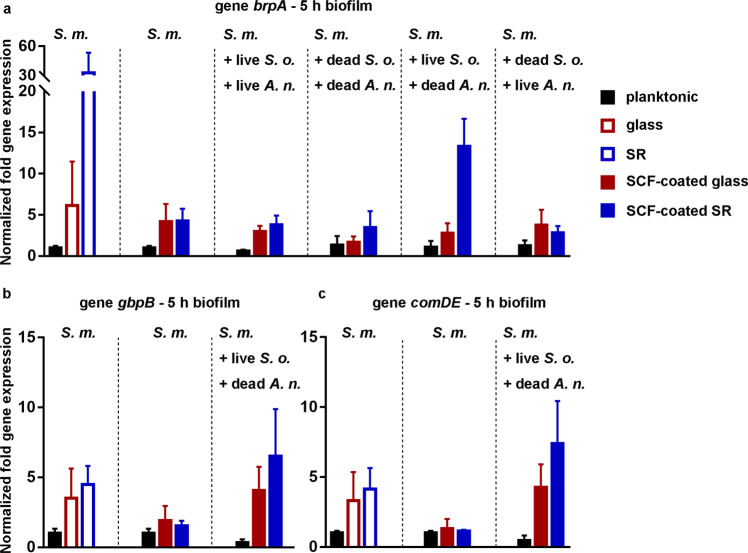
Gene expression in *S. mutans* UA159 mono-species biofilms and different triple-species oral biofilms. Different triple-species biofilms were comprised of *S. mutans* UA159 (*S. m*.) in combination with live or dead *S. oralis* J22 (*S. o*.) and *A. naeslundii* T14V-J1 (*A. n*.) in the absence and presence of an adsorbed salivary-conditioning film on different substratum surfaces. **a** *brpA* gene expression. **b** *gbpB* gene expression. **c** *comDE* gene expression. Error bars represent SDs over triplicate experiments with separately grown biofilms. Data on *brpA*, *gbpB*, and *comDE* expressions in *S. mutans* UA159 mono-species biofilms on glass and silicone rubber surfaces in the absence of an adsorbed salivary-conditioning film were taken from Wang et al.^8^.

Adaptive gene expression of *brpA*, *gbpB*, and *comDE* in *S. mutans* UA159 biofilms, previously demonstrated to be most adhesion force sensitive genes^8^ was determined in planktonic and mono-, dual-, and triple-species biofilms with RT-qPCR. In the absence of an adsorbed SCF, 5 h *S. mutans* mono-species biofilm displayed *brpA* gene expression that increased from a planktonic state to an adhering state. Expression of *brpA* gene was higher on hydrophobic silicone rubber than on hydrophilic glass (Fig. 3a). Once coated with a SCF, differences in *brpA* gene expression in *S. mutans* biofilms on both substratum surfaces completely disappeared, but expression remained higher than for *S. mutans* in a planktonic state. This typical ranking of *brpA* gene expression observed in *S. mutans* mono-species biofilms in the absence of an adsorbed SCF was nearly fully restored in triple-species biofilms containing live *S. mutans* and *S. oralis* in combination with dead *A. naeslundii*. Triple-species biofilms in which all three strains were alive suggest the on-set of restoration of the ranking observed for *S. mutans* mono-species biofilms, but not as convincingly as when dead *A. naeslunddii* were included in the biofilm.

Restoration of the ranking as observed for *brpA* gene expression in *S. mutans* mono-species biofilms in the absence of an adsorbed salivary film on the different surfaces by live *S. oralis* was confirmed in triple-species biofilms for *gbpB* (Fig. 3b) and *comDE* (Fig. 3c) gene expression on SCF-coated surfaces. Differences in gene expressions were minor in 24 h mono-species and triple-species oral biofilms (Supplementary Fig. 3).

## Discussion

In this study, we aimed to determine whether the principles of mechano-microbiology^2^, most notably adhesion force sensing^21^ with respect to environmental sensing by bacteria adhering to substratum surfaces, are preserved in multi-species biofilms in the absence and presence of an adsorbed SCF on the surfaces. The early dominance of *S. oralis* in 5 h-old triple-species biofilms is followed by *S. mutans* dominance in 24 h-old biofilms. In the absence of the ability to co-aggregate with the two initial colonizers involved in this study (see Table 2), *S. mutans* appears later in spatio-temporal sequence of multi-species biofilm growth in vitro^22^ and oral biofilm formation in general^13^ due to the acid environment it needs to create for itself^15,16^.

Hydrophobicity is an important substratum surface characteristic determining bacterial adhesion forces. Generally, bacterial adhesion forces are stronger on hydrophobic surfaces than on more hydrophilic surfaces^23,24^. In an environment, laden with proteins, protein adsorption proceeds faster than bacterial adhesion because proteins diffuse faster towards a surface than bacteria^25,26^ Hence, in many environments, bacteria adhere to a protein film-coated surface^27^. Saliva is composed of a large variety of proteins that maintain surface thermodynamic homeostasis^28,29^ and converge differences in the hydrophobicity of surfaces exposed to the oral cavity to a well-controlled, narrow range. Oral surface thermodynamic homeostasis is achieved by polar–apolar layering in SCF adsorption^28^, i.e., hydrophilic surfaces, such as a glass surface, are made more hydrophobic by allowing protein adsorption in a conformation that presents hydrophobic groups to its outer surface, while on a hydrophobic surface the opposite occurs. Polar–apolar layering therewith explains why stationary adhesion forces sensed by *S. mutans* decreased on hydrophobic silicone rubber and increased on hydrophilic glass upon SCF coating to a similar value (Table 1). Oral surface thermodynamic homeostasis therewith fully explains why *S. mutans* did not display differential adhesion force sensing in 5 h mono-species biofilms on SCF-coated surfaces (Fig. 3).

Having excluded differential adhesion force sensing on different SCF-coated materials surfaces, we now address the probability of *S. mutans* sensing of adhesion forces arising from other species in our triple-species biofilms. The probability that *S. mutans* also senses adhesion forces arising from other bacteria is considered low, as co-aggregation between *S. mutans* and any of the two initial colonizers involved in this study, was absent (Table 2). Also, volumetric bacterial densities of different biofilms in the absence of co-aggregating strains and species are generally^30^ <0.3 bacteria/µm^3^ with distances between biofilm inhabitants between 1 and 3 µm^31^. This is far beyond the range (<500 nm) of adhesion forces between surfaces^32,33^ and excludes interferences in *S. mutans* adhesion force sensing by direct contact with other bacteria in a multi-species biofilm.

The ranking of gene expression by planktonic *S. mutans* and *S. mutans* adhering on substratum surfaces in the absence of a SCF was only restored in triple-species biofilms with live *S. oralis* present (see also Fig. 3). *S. mutans* gene expression in the presence of live *S. oralis* was weaker in the additional presence of live *A. naeslundii* than in the presence of dead *A. naeslundii* on SCF-coated silicone rubber, presumably because both initial colonizers in a live state competed for real estate of a SCF-coated substratum. Having now excluded *S. mutans* sensing of adhesion forces arising from different SCF-coated materials and from other bacteria in our triple-species biofilms, this leaves the question how *S. mutans* adhesion force sensing on SCF-coated surfaces is restored to the ranking observed on uncoated materials surfaces.

To answer this question, we turn to the sheet detachment observed. Sheet detachment of oral biofilm from SCF-coated substrata is a new phenomenon to us, which may have remained unnoticed, also in the literature, because the field of view of microscopic observation applied in most studies is much smaller than in OCT (~10 mm^2^ in OCT as applied in this study vs. 0.1 mm^2^, e.g., in confocal laser scanning microscopy (CLSM) at ×40 magnification). Also, the search for beautiful images as common in microscopic observation may have biased the literature and prevented observation of sheet detachment of biofilm. Sheet detachment from SCF-coated surfaces of our *S. mutans* mono-species and *S. mutans* containing triple-species biofilms exclusively occurred in the absence of live *S. oralis*. Oral bacteria adhere significantly with weaker forces to SCF-coated surfaces as compared with surfaces in the absence of SCFs^20,34^. It may be considered a new, befitting role for initial colonizers to facilitate direct adhesion of other biofilm inhabitants to a substratum surface and therewith establish much stronger adhesion forces of bacteria with a substratum surface than established when the SCF is in between. *S. oralis*, with its high prevalence in triple-species biofilms as opposed to *A. naeslundii* (see Fig. 2), is ideally equipped for enzymatic degradation of salivary proteins. *S. oralis* is known to possess glycosidase and proteolytic activities to break down SCF components into metabolizable fragments^35^. Oppositely and in addition to its low prevalence, *A. naeslundii* is not able to degrade oral mucins, unless in combination with *Streptococcus mitis*, *Streptococcus gordonii*, and *Streptococcus cristatus*^36^. Collectively, these considerations lead to the conclusion that *S. oralis* clears the way through a salivary condition film for strong adhesion forces of a biofilm and direct contact of other biofilm inhabitants to directly contact a surface. This allows *S. mutans* surface sensing to define its appropriate response to SCF-coated substratum surfaces in multi-species biofilms.

Adhesion force sensing was not observed in 24 h triple-species biofilms, nor in mono-species *S. mutans* biofilms regardless of the absence or presence of an adsorbed SCF on a substratum surface (Supplementary Fig. 3). Previously, we attributed this to the limited calling distance of quorum sensing^8^. Analysis of gene expression of cross-sections of mono-species *S. mutans* biofilms on substrata in the absence of a SCF demonstrated the extension of adhesion force-induced gene expressions up to a height of 20–30 µm above a surface, far above the thickness of our 24 h triple-species biofilms (Fig. 1).

In summary, bacteria cause infections by adhering to a variety of protein coated natural or synthetic surfaces in the human body^37,38^. Most human infections are caused by bacteria in a biofilm mode of growth, in which they surface adapt^39^. This study shows that in multi-species oral biofilms adhering to SCF-coated surfaces, surface adaptation by *S. mutans* to surfaces remains to exist. Based on available literature, this is suggested to occur through breaking down of the SCF by initial colonizers, most notably *S. oralis*. The three genes considered in this article to establish this conclusion (gene *brpA*, *gbpB* and *comDE*) are all relevant for the production of the *S. mutans* protective extracellular matrix^40–42^. As *S. mutans* is a prominent cariogenic pathogen^43^ whose colonization of the oral cavity may initiate serious organ infection^44^ including endocarditis^45^, the importance of this conclusion extends far beyond oral health.

## Methods

### Substratum materials and SCF adsorption

Glass (Thermo Scientific, Braunschweig, Germany) and medical-grade silicone rubber (ATOS Medical B.V., Zoetermeer, The Netherlands) were used as substrata, as they have been previously shown to represent two ends of the scale with respect to hydrophobicity (water contact angles 11° and 103°, respectively) and *S. mutans* gene expression (*brpA* gene expression was maximal on hydrophobic medical-grade silicone rubber surface and minimal on hydrophilic glass surface) in mono-species biofilms^8^ All materials were made to fit into a 24-well plate, allowing samples with a surface area of 1 cm^2^, cleaned with 2% RBS 35 detergent (Omnilabo International BV, The Netherlands) under sonication for 5 min, rinsed with warm tap water, sterilized with 70% ethanol, and finally washed with sterilized demineralized water.

Depending on the experiment, cleaned surfaces were coated with a SCF. To this end, human whole saliva was collected and prepared, as previously described^27,46^. Briefly, human whole saliva from 20 healthy volunteers of both sexes was collected into ice-chilled Erlenmeyer flasks after stimulation by chewing Parafilm^®^. Saliva was collected with the informed consent of the voluntary saliva-donors, in accordance with the rules set out by the Ethics Committee at the University Medical Center Groningen. After collection, the salivary samples were pooled and centrifuged twice (10,000 × *g*, 15 min, 4 °C), and phenylmethylsulfonyl fluoride was added to a final concentration of 1 mM as a protease inhibitor. Afterwards, the solution was centrifuged again, dialyzed (24 h, 4 °C) against demineralized water and freeze-dried for storage. For SCF adsorption, freeze-dried saliva was dissolved in a calcium-phosphate buffer (1 mM CaCl_2_, 2 mM potassium phosphate, 50 mM KCl pH 6.8) at a concentration of 1.5 mg/ml and samples were immersed in 1 ml of reconstituted human whole saliva for overnight adsorption. After SCF adsorption, samples were immediately used for further experiments.

### Bacterial strains, growth conditions, and harvesting

All bacterial strains used were cultured on blood agar (Mediaproducts BV, Groningen, The Netherlands) plates from a frozen stock. *S. mutans* UA159 was grown at 37 °C in 5% CO_2_ for 24 h, *S. oralis* J22 at 37 °C in ambient air for 24 h, and *A. naeslundii* T14V-J1 at 37 °C under anaerobic conditions for 48 h. Next, one colony of each strain was inoculated in 10 ml brain heart infusion (BHI) broth (Oxoid, Basingstoke, UK) with 1% (wt/vol) sucrose added and cultured at 37 °C in 5% CO_2_ for 24 h. These pre-cultures were used to inoculate main cultures (1 : 20 dilution), which were grown for 16 h. Bacteria were collected by centrifugation (Beckman J2-MC centrifuge; Beckman Coulter, Inc., Pasadena, CA, USA) for 5 min at 5000 × *g* and washed twice in buffer. To break streptococcal chains, bacterial suspensions were sonicated once for 30 s at 130 W (Vibra cell model VCX130, Newtown, Connecticut, USA) while cooling in an ice-water bath. The bacterial suspensions were diluted in buffer to a concentration appropriate for the respective experiments, as determined by enumeration in a Bürker-Türk counting chamber. Non-viable *S. oralis* J22 and *A. naeslundii* T14V-J1 suspension were prepared by heat killing of the suspended bacteria in a water bath, kept at 60 °C for 30 min. Heat killing was confirmed using plate counting (see below).

### Bacterial cell surface characterization

Zeta potentials are an important characteristic of bacteria, relevant for initial adhesion^32^ and were determined in bacterial suspensions (3 × 10^8^/ml) in the above described calcium-phosphate buffer using particulate microelectrophoresis (Zetasizer nano-ZS; Malvern Instruments, Worcestershire, UK) at 37 °C. Zeta potentials of all strains were measured in triplicate with different bacterial cultures, and data are presented as averages ± SDs of the mean.

### Co-aggregation assay

Pairwise co-aggregation properties of the three bacterial strains involved in the biofilm model applied were measured in order to determine whether or not bacteria in different combinations had direct adhesive contact with each other. For co-aggregation measurements, we have used the well-established and generally-accepted, semi-quantitative Kolenbrander assay^19^. Co-aggregation was assayed by mixing equal volumes (0.5 ml) of one or more bacterial suspensions (1 × 10^9^/ml) in buffer during 120 min and subsequently monitoring the decrease in turbidity of the suspension due to co-aggregation. After mixing, co-aggregation was scored on a scale from 0 (evenly turbid suspension) to 4 (fully clear suspension)^19^. A full description of all scores is given in the footnote to Table 2 and visual images of each score are presented in Supplementary Fig. 4.

### Adhesion force measurements

For adhesion force measurements, single bacterial contact probes^47^ were prepared as described before^7,48^. First, *S. mutans* was attached to a tipless cantilever (NP-O10; Bruker AFM Probes, Camarillo, CA, USA) via electrostatic double-layer interaction with poly-l-lysine (PLL) (molecular weight 70,000 to 150,000; Sigma-Aldrich, St. Louis, MO, USA) adsorbed to the cantilever using a micromanipulator (Narishige Groups, Tokyo, Japan). Cantilevers were calibrated using the thermal method, yielding spring constants in the range of 0.03 to 0.12 N/m. Briefly, the far end of a tipless cantilever was dipped in a droplet of PLL for 1 min and dried in air for 2 min, followed by 2 min of immersion in a droplet of a streptococcal suspension at a relatively low concentration (3 × 10^7^/ml in buffer) to allow one bacterium to adhere to the cantilever. Single bacterial adhesion from low concentration suspensions was demonstrated previously using fluorescence microscopy of LIVE/DEAD stained (Baclight viability stain; Molecular Probes Europe BV, Leiden, The Netherlands) Staphylococci^7^ and Streptococci^48^ attached to a PLL-coated cantilever. Use of LIVE/DEAD staining furthermore allowed us to conclude that the viability of bacteria was not affected by adhesion to a PLL-coated cantilever, as also demonstrated by others for *Escherichia coli*^49^. For the inadvertent event that multiple bacteria might have adhered to a cantilever, bacterial probes were used for imaging. All bacterial probes made in the above way, correctly imaged and reflected the dimensions of AFM calibration grids^7,48^ without the occurrence of double-contour lines indicative of double-contact. *S. mutans* probes in the current study were checked for single bacterial adhesion to the cantilever (Supplementary Fig. 5a) and the absence of double-contour lines generation by imaging streptococci adhering to a glass surface (Supplementary Fig. 5b). Freshly prepared streptococcal probes were directly used for adhesion force measurements. Adhesion force measurements were performed at room temperature in the calcium-phosphate buffer using a Dimension 3100 system (Nanoscope V; Digital Instruments, Woodbury, NY, USA). Adhesion forces were measured on glass and silicone rubber surfaces with and without a SCF and bacterial cell surfaces. Briefly, the cantilever with an attached *S. mutans* was brought into contact with a substratum surface or a bacterial cell surface and retracted after a specified time (bond maturation time). Upon retraction, the cantilever bends until the bacterial bond with the surface under probing was disrupted. The force at which this occurred was subsequently calculated from the cantilever bending and recorded as the adhesion force of the bacterium to the surface probed. For each bacterial probe, force–distance curves were measured after 0, 2, 5, 10, and 30 s of bond maturation time at a 5 nN loading force. To verify whether a measurement series had disrupted bacterial integrity, five force–distance curves at 0 s bond maturation time were measured at the beginning and end of each experiment on glass. When the adhesion forces measured differed >1 nN from the beginning to the end of an experiment, data were discarded and the bacterial probe was replaced by a new one.

Single bacterial probes were directly used on the different substratum surfaces to measure the adhesion force between the bacterium and the surface in the absence and presence of a SCF. *S. mutans* probes were in addition used to measure its adhesion forces with *S. oralis* or *A. naeslundii*. To this end, *S. oralis* or *A. naeslundii* were immobilized on glass slides, in essence similarly as described above for the preparation of a streptococcal probe. In short, a droplet of PLL was adsorbed to a cleaned glass surface and dried in air, after which a droplet of a bacterial suspension (3 × 10^8^/ml) was pipetted onto the surfaces and dried in air for another 15 min. Surfaces were rinsed with demineralized water and dried in air for 15 min, resulting in partly dehydrated bacteria on the surface by the loss of free water from its surface^50^ without removal of internally bound water. This type of drying did not affect bacterial viability^7,48^ as can also be seen from the green fluorescence of the single LIVE/DEAD stained bacterium on’ the cantilever in Supplementary Fig. 5a. Before adhesion force measurements, the surfaces were scanned using the AFM and zoomed in to ensure the contact between the *S. mutans* probe and a single target bacterium (either *S. oralis* or *A. naeslundii*) attached to the surface.

### Triple-species biofilm formation

Bacterial suspensions of the three bacterial strains were mixed (1 : 1 : 1 volume) to provide an inoculum with a defined final concentration of *S. mutans* (1 × 10^8^/ml), *A. naeslundii* (either live or dead, 1 × 10^9^/ml), and *S. oralis* (either live or dead, 1 × 10^8^/ml).

To grow a triple-species biofilm, 1 ml of the mixed bacterial suspension was added to a 24-well plate containing a glass or silicone rubber sample with an adsorbed SCF. Wells were kept under static conditions for 2 h at 37 °C in 5% CO_2_ to allow bacterial sedimentation and adhesion. After 2 h, the bacterial suspension was removed, and each well was carefully washed once with 1 ml buffer, after which 1 ml BHI complemented with 1% (wt/vol) sucrose was added to each well to allow biofilm growth under static conditions in 5% CO_2_ at 37 °C. After 5 or 24 h of growth, all biofilms were carefully washed with buffer and imaged using OCT, to determine their thicknesses (see below). Subsequently, biofilms were carefully scraped off the surfaces and resuspended in buffer for plate enumeration of colony forming units (CFUs) or for RNA isolation and gene expression (see below).

### Enumeration of CFUs

For enumeration of the number of CFUs in different biofilms, bacteria were scraped off the different surfaces, suspended in buffer, tenfold serially diluted and agar plated. Selective agar plates were used to differentiate between the different strains isolated from triple-species biofilms. Home-made TSY20B agar^51^ plates were used for enumeration of *S. mutans*, commercially-bought blood agar plates were used for enumeration of *S. oralis* and home-made CFAT^52^ agar plates for *A. naeslundii* enumeration. Briefly, 100 µl of a bacterial dilution was pipetted onto the corresponding agar plate, spread using a glass swab and left to incubate under different conditions. Blood agar plates were kept in ambient air at 37 °C for 24 h, TSY20B agar plates in 5% CO_2_ at 37 °C for 48 h, and another 24 h in ambient air and CFAT agar plates were left at 37 °C under anaerobic conditions for 4 days. Bacterial colonies were enumerated using a plate counter (New Brunswick Scientific Edison, NJ, USA) after incubation.

### Optical coherence tomography

Biofilm thicknesses were measured using OCT (Ganymede II, Thorlabs Ganymede, Newton, NJ, USA) equipped with a 930 nm center wavelength white light beam and a Thorlabs LSM03 objective scan lens. The imaging frequency was 30 kHz, with a sensitivity of 101 dB, and the refractive index was set as 1.33 for the biofilm, equal to the one of water. Two-dimensional images had fixed 5000 pixels with variable pixel size, while containing a variable number of pixels with a 2.68 µm pixel size in the vertical direction. Images were created by the OCT software (Thor-Image OCT 4.1) using 32 bit data and signal intensities of back-scattered light were reflected by a whiteness distribution in OCT images. Biofilm thickness was subsequently determined from the OCT images after thresholding as described by Otsu^53^.

### Gene expression of planktonic and biofilm-grown bacteria

Planktonic as well as resuspended biofilm bacteria were centrifuged at 6500 × *g* for 5 min, supernatants removed, and pellets stored at −80 °C until RNA isolation. Total RNA was isolated using RiboPure bacterial kit (Ambion, Invitrogen, Foster City, CA) according to the manufacturer’s instructions. Traces of genomic DNA were removed using the DNAfree kit (Ambion, Applied Biosystems, Foster City, CA, USA). The amount and quality of extracted RNA was based on the 260/280-nm ratio measured using a NanoDrop ND-1000 (NanoDrop Technologies LLC, Thermo Fisher Scientific, Wilmington, DE, USA). A ratio of A260/A280 absorbance of around 2.0 (±10%) was accepted as “pure” for RNA. A mixture of 200 ng RNA, 4 µl 5× iScript reaction mixture, and 1 µl iScript reverse transcriptase, in a total volume of 20 µl (Iscript; Bio-Rad, Hercules, CA, USA), was used for cDNA synthesis according to the manufacturer’s instructions. Real-time RT-qPCR was performed in a 384-well plate (HSP-3905; Bio-Rad Laboratories, Foster City, CA, USA) with the primer sets for the selected genes (Supplementary Table 2). The following thermal conditions were used for all RT-qPCRs: 95 °C for 3 min and 39 cycles of 95 °C for 10 s, and 59 °C for 30 s. The mRNA levels were quantified in relation to endogenous control gene coding for 16S *rRNA*. Gene expression levels in the biofilms were normalized to planktonic *S. mutans* UA159. Gene expression was assessed in triplicate experiments with separately grown cultures. Specific primers were chosen for analyzing gene expression of *S. mutans*. None of the primers presents cross-reaction between streptococci and actinomyces or between different streptococcal strains^54^ as independently confirmed for our triple-species biofilms.

### Statistical analysis

GraphPad Prism, version 7 (San Diego, CA, USA), was employed for statistical analysis. Significant differences between groups were assessed by one-way ANOVA followed by Dunn’s multiple-comparison test. Alternatively, the Mann–Whitney test was used to compare two sets of data at a time. Significance was adapted at *P* < 0.05.

### Reporting summary

Further information on research design is available in the Nature Research Reporting Summary linked to this article.

## Supplementary information

Supplementary Information

Reporting Summary

## Data Availability

All data have been used in the paper and raw data are available upon request.
