# Supplementary material for: Streptococcus mutans adhesion force sensing in multi-species oral biofilms
Source: NPJ Biofilms Microbiomes. 2020 Jun 24;6:25. doi: 10.1038/s41522-020-0135-0 (PMC7314845; doi:10.1038/s41522-020-0135-0)
Supplement: Supplementary file 1 — Supplementary Information [file 41522_2020_135_MOESM1_ESM.pdf]

Supplementary Material

Streptococcus mutans adhesion force sensing in multi-species oral biofilms

Can Wang,[a] Henny C. van der Mei,[b] Henk J. Busscher,[b] Yijin Ren[a]

[a]University of Groningen and University Medical Center Groningen, W.J. Kolff Institute, Department of Orthodontics, Groningen, The Netherlands.

[b]University of Groningen and University Medical Center Groningen, W.J. Kolff Institute, Department of Biomedical Engineering, Groningen, The Netherlands.

#Address correspondence to H.C. van der Mei. Email: h.c.van.der.mei@umcg.nl

Supplementary Table 1 Zeta potentials measured in a calcium-phosphate buffer (1 mM $CaCl_2$, 2 mM potassium phosphate, 50 mM KCl, pH 6.8) of the three bacterial strains involved in this study, demonstrating similar zeta potentials of live and dead *S. oralis* J22 and *A. naeslundii* T14V-J1 (no significant difference at $P > 0.05$, Mann-Whitney test). ± signs represent standard deviations (SD) over triplicate experiments.

**Strains**	**Live/dead**	**Zeta potential (mV)**
S. mutans UA159	live	-2.8 ± 0.3
S. oralis J22	live	-8.4 ± 0.2
	dead	-8.7 ± 0.5
A. naeslundii T14V-J1	live	-4.6 ± 0.3
	dead	-5.3 ± 0.5

Supplementary Table 2 Primer sequences of the genes used in this study.

Gene	Primer sequence (5′ to 3′)	
	forward	reverse
16S rRNA	ACCAGAAAGGGACGGCTAAC	TAGCCTTTTACTCCAGACTTTCCTG
brpA	GGAGGAGCTGCATCAGGATTC	AACTCCAGCACATCCAGCAAG
gbpB	ATACGATTCAAGGACAAGTAAG	TGACCCAAAGTAGCAGAC
comDE	ACAATTCCTTGAGTTCCATCCAAG	TGGTCTGCTGCCTGTTGC

Calculation of initial adhesion forces, characteristic bond maturation times and stationary adhesion forces

Adhesion forces have been described[1] to increase with contact time between the interacting according to an exponential function

$$\mathrm{F_t} = \mathrm{F_0} + (\mathrm{F_{stationary}} - \mathrm{F_0})(\exp(-\frac{t}{\tau})) \quad (1)$$

in which F_t is the adhesion force after bond maturation time t, F_0 is the initial adhesion force at 0 s bond maturation time and $F_{stationary}$ indicates the stationary adhesion force reached after complete bond maturation, while τ is the characteristic time constant for bond maturation. Least-square fitting of F_0, τ and $F_{stationary}$ to the adhesion forces measured as a function of time was applied for their calculation.

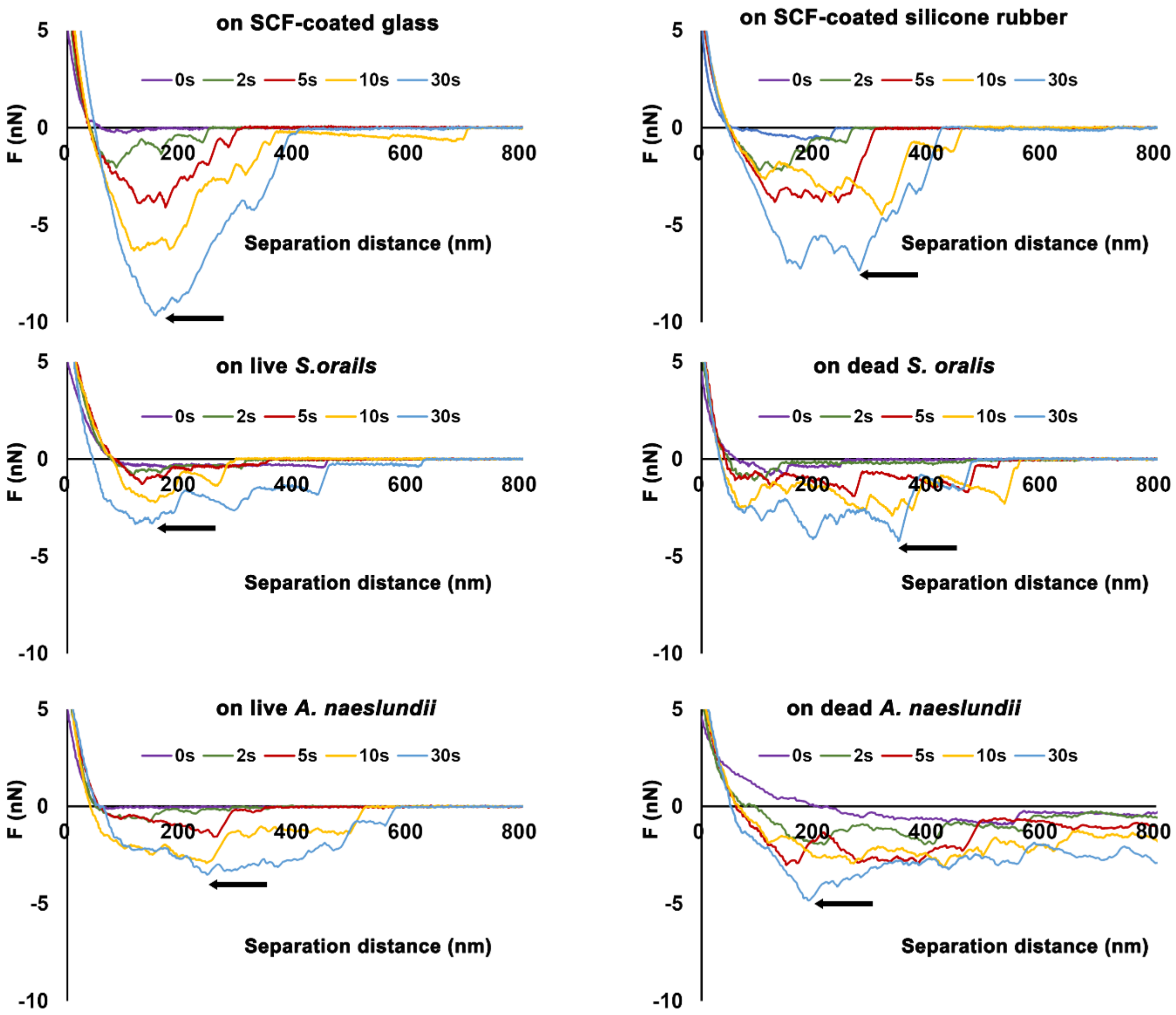

Supplementary Fig. 1 Examples of retraction force-distance curves taken after different bond maturation times for a *S. mutans* UA159 probe on SCF-coated glass (left) and SCF-coated silicone rubber (right) surfaces, on live *S. oralis* J22 and *A. naeslundii* T14V-J1 cell surfaces (left) and on dead *S. oralis* J22 and *A. naeslundii* T14V-J1 cell surfaces (right). The arrow points to the most-negative force value, taken as the adhesion force for a given curve.

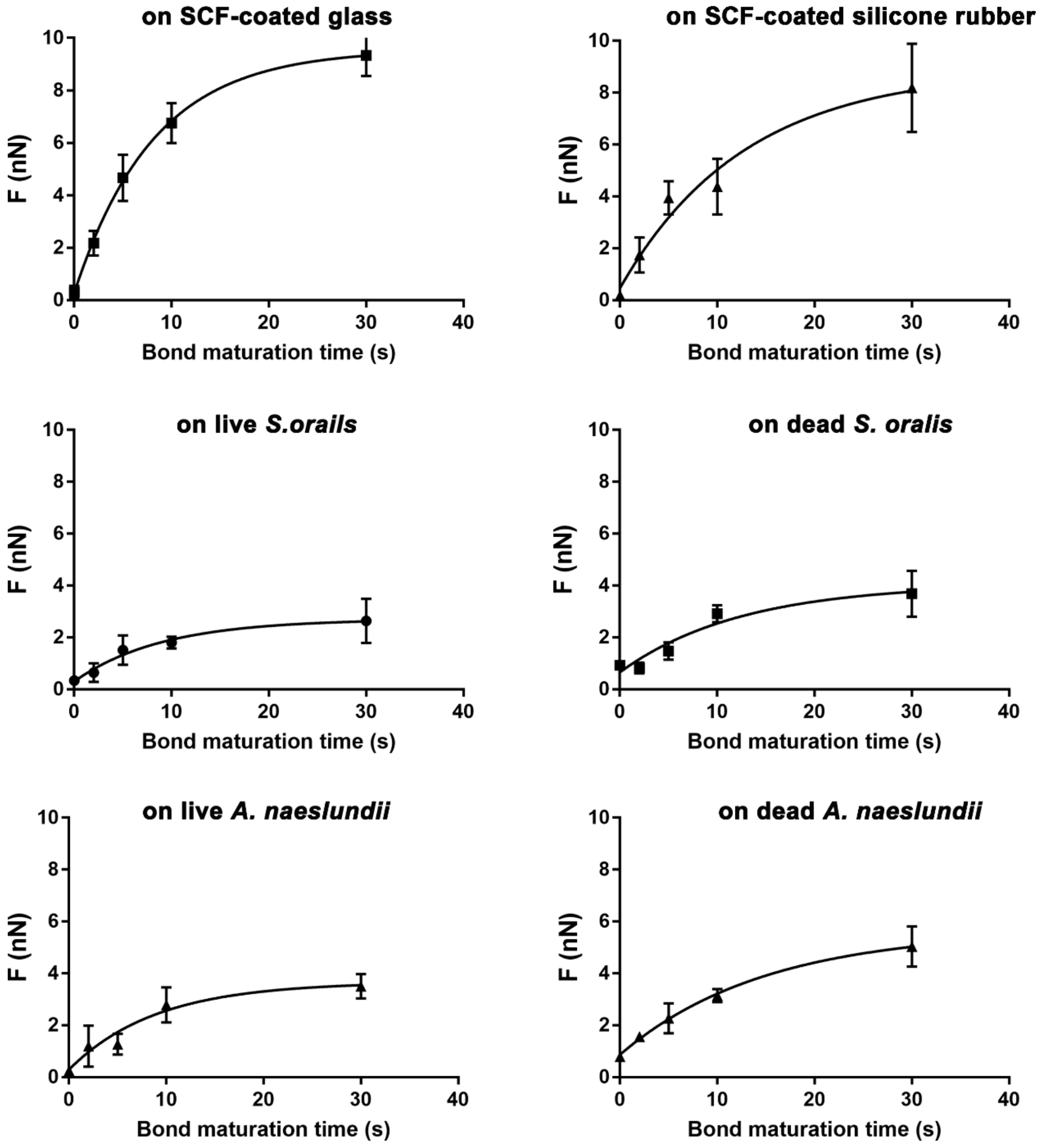

Supplementary Fig. 2 *S. mutans* UA159 adhesion forces as a function of bond maturation time on SCF-coated glass (left) and SCF-coated silicone rubber (right) surfaces, on live *S. oralis* J22 and *A. naeslundii* T14V-J1 cell surfaces (left) and on dead *S. oralis* J22 and *A. naeslundii* T14V-J1 cell surfaces (right). Error bars represent standard deviations (SD) over example measurements, each taken on five contact points with one *S. mutans* probe.

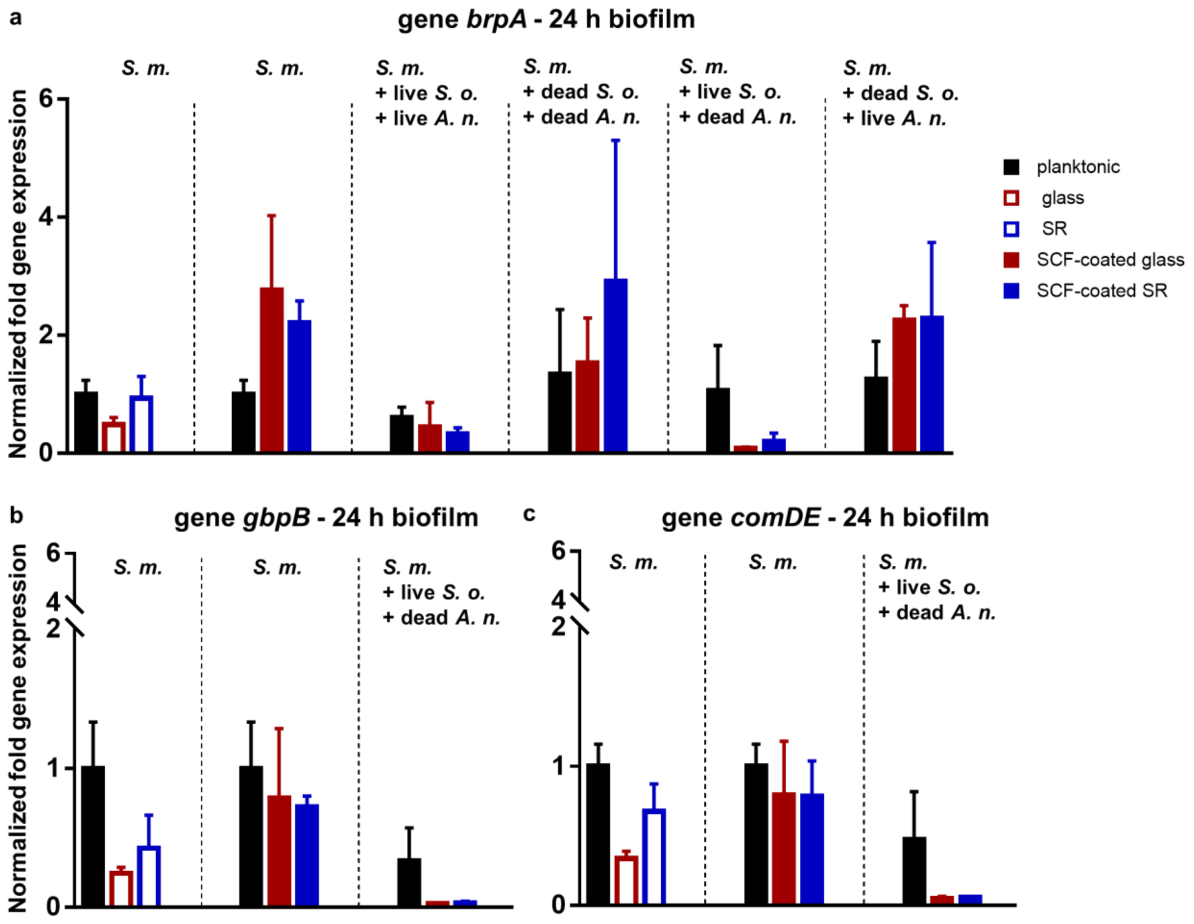

Supplementary Fig. 3 Gene expression in 24 h *S. mutans* UA159 (*S. m*.) mono-species biofilms and different triple-species oral biofilms. Biofilms comprised of *S. mutans* UA159 (*S. m.*) in combination with live or dead *S. oralis* J22 (*S. o*.) and *A. naeslundii* T14V-J1 (*A. n*.) on different substratum surfaces in the absence and presence of a salivary conditioning film.

a *brpA* gene expression

b *gbpB* gene expression

c *comDE* gene expression.

Error bars represent SD values over triplicate experiments with separately grown biofilms. Data on *brpA*, *gbpB* and *comDE* expressions in *S. mutans* UA159 mono-species biofilms on glass and silicone rubber surfaces in absence of a salivary conditioning film were taken from Wang et al.[2]

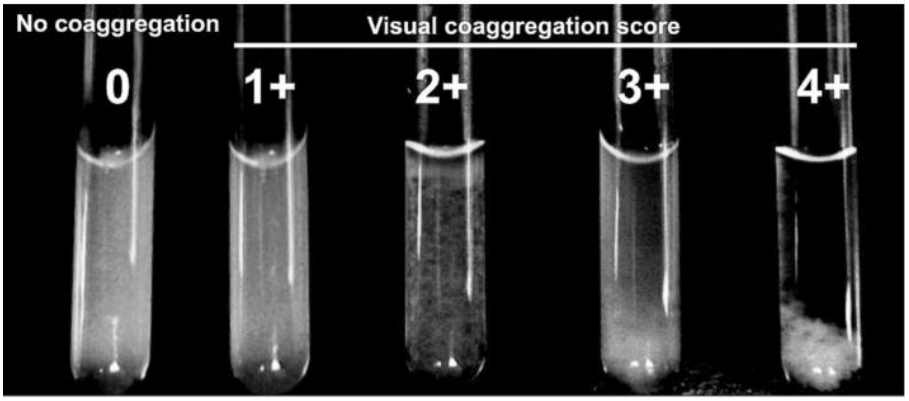

Supplementary Fig. 4 Visual aggregation assay showing different coaggregation scores. Score 0: no change in turbidity and no visible co-aggregates; Score 1: weak coaggregation with dispersed aggregates in a turbid background; Score 2: clearly visible, small co-aggregates, not settling immediately; Score 3: large settling co-aggregates, leaving a slightly turbid suspension; Score 4: maximum coaggregation large co-aggregates settled immediately leaving a fully clear supernatant.(Taken from K. R. Min, M. N. Zimmer & A. H. Rickard (2010) Physicochemical parameters influencing coaggregation between the freshwater bacteria *Sphingomonas natatoria* 2.1 and *Micrococcus luteus* 2.13, Biofouling, 26:8, 931-940, DOI: 10.1080/08927014.2010.531128 and reprinted with permission from Taylor & Francis Ltd., https://pubmed.ncbi.nlm.nih.gov/21058055).

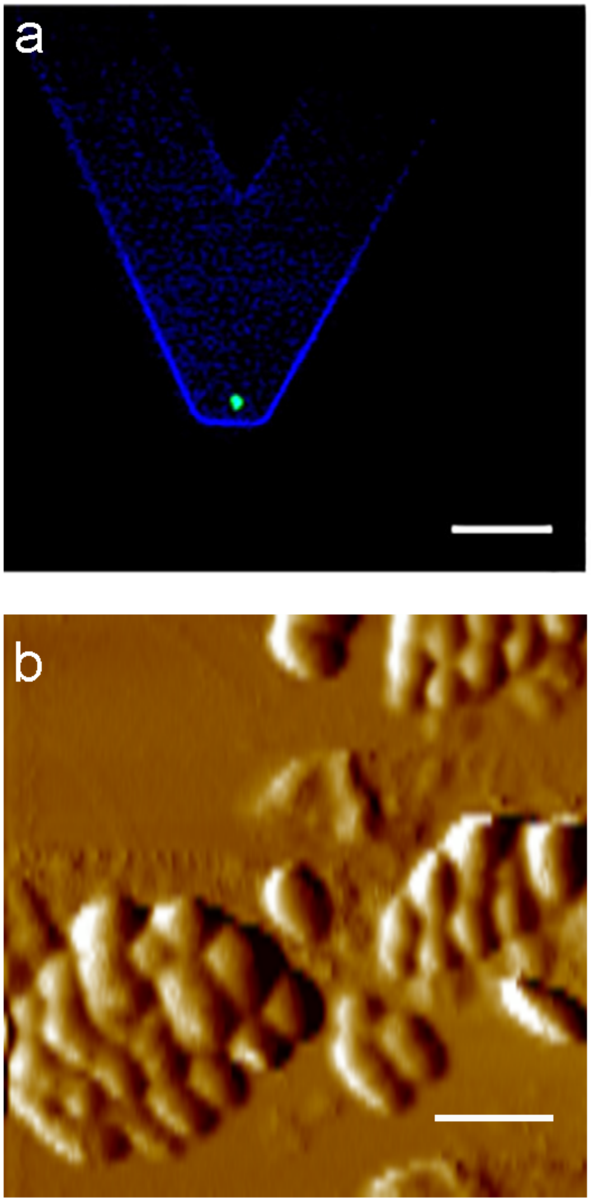

Supplementary Fig. 5 Demonstration of single bacterial probe use.

a. Fluorescence image of a single LIVE/DEAD stained *S. mutans* UA159 bacterium adhering on a tipless AFM cantilever. Green fluorescence demonstrates bacterial viability. Scale bar represents 20 μm.

b. Verification of the absence of double-contour lines upon imaging *S. oralis* adhering to a glass surface, using a *S. mutans* probe. Scale bar represents 2 μm.

References

1. Busscher, H. J., Norde, W., Sharma, P. K. & Van der Mei, H. C. Interfacial re-arrangement in initial microbial adhesion to surfaces. *Curr. Opin. Colloid. Interf. Sci.* **15,** 510-517 (2010).
2. Wang, C., Hou, J., Van der Mei, H. C., Busscher, H. J. & Ren, Y. Emergent properties in *Streptococcus mutans* biofilms are controlled through adhesion force sensing by initial colonizers. *mBio* **10,** e01908-19 (2019).
